# Partial Replacement of Municipal Incinerated Bottom Ash and PET Pellets as Fine Aggregate in Cement Mortars

**DOI:** 10.3390/polym14132597

**Published:** 2022-06-27

**Authors:** Lalitsuda Phutthimethakul, Nuta Supakata

**Affiliations:** 1 International Program in Hazardous Substance and Environmental Management, Graduate School, Chulalongkorn University, Bangkok 10330, Thailand; adustilal@gmail.com; 2 Department of Environmental Science, Faculty of Science, Chulalongkorn University, Bangkok 10330, Thailand; 3 Research Group (STAR): Waste Utilization and Ecological Risk Assessment, The Ratchadaphiseksomphot Endowment Fund, Chulalongkorn University, Bangkok 10330, Thailand

**Keywords:** cement mortars, municipal incinerated bottom ash, PET pellets

## Abstract

The objective of this study was to examine the optimal mixing ratio of municipal incinerated bottom ash (MIBA) and PET pellets used as a partial replacement of fine aggregates in the manufacture of cement mortars. As a partial replacement for sand, 15 mortar specimens were prepared by mixing 0%, 10%, 20%, 30%, and 40% municipal incinerated bottom ash (MIBA) (A) and 0%, 10%, and 20% PET pellets (P) in 5 cm × 5 cm × 5 cm cube molds. The cement/aggregate ratio was 1:3, and the water/cement ratio was 0.5 for all specimens. The results showed that the compressive strength of cement mortars decreased when increasing the amount of MIBA and PET pellets. The mortar specimens with 10% PET pellets achieved the highest compressive strength (49.53 MPa), whereas the mortar specimens with 40% MIBA and 20% PET pellets achieved the lowest compressive strength (24.44 MPa). Based on this finding, replacing 10% and 20% sand in cement mortar with only MIBA or only PET pellets could result in compressive strengths ranging from 46.00 MPa to 49.53 MPa.

## 1. Introduction

In 2018, cities around the world generated approximately two billion tons of solid waste [1]. Improper disposal, such as landfilling and incineration, has resulted in the release of toxic elements and pollutants that contaminate air, water, and soil and endanger human health [2,3]. Waste recycling contributes to decoupling economic growth from resource use [4,5,6] while reducing emissions of greenhouse gases and pollutants.

The waste management problem in Si Chang Island is a long-standing problem. The main reasons are geographic problems such as rocky areas, a lack of appropriate technology, and a lack of funds. Most of the waste is treated by incineration, which generates municipal incinerated bottom ash (MIBA) that is not properly managed. Recyclable waste is also difficult to manage as it needs to be transported to recycling facilities on the shore. One type of recycled waste is PET bottles, which are imported and consumed in large quantities [7,8].

The waste composition at the Si Chang disposal site in 2020 showed food waste as the most significant component (43.46%), followed by glass waste (22.25%) and plastic waste (12.35%). Some recycling wastes were sorted before incineration, but most waste is still incinerated without complete separation. Since incinerators have a low efficiency, they can eliminate around 78% of the waste collected daily. The incineration residue consists of mixed ash (municipal incinerated bottom ash, MIBA), which is approximately 2000 kg per month and is disposed of in dumpsites with the potential to contaminate the environment [9,10]. Many studies have shown that the chemical composition of ash depends on the types of waste. They also investigated the possibilities of utilizing ash as fine and coarse aggregate in various applications such as mortar, concrete, road pavements, masonry blocks, lightweight concrete, and foamed concrete [10,11,12]. Therefore, this study was interested in using MIBA as a partial replacement for fine aggregates.

Data from the 2017 to 2019 waste bank projects operated by Si Chang municipality confirm that PET bottle waste is the most common type of waste compared to other plastic waste, at approximately 635 kg per year [13]. Its current management is to store it for approximately 3–6 months and send it by marine transport to a recycling facility on the mainland. However, PET bottles require more space for storage due to their low density, causing higher management costs [8]. Therefore, finding a solution for handling PET plastic bottle waste on the island is an exciting alternative.

Population growth and tourism development have increased the demand for construction. As a result, construction materials have become more expensive due to the scarcity of natural resources and rising transportation costs [14]. Numerous studies have been conducted on the compressive strength of cement mortars made from mixed materials [15,16,17]. Instead of dumping PET waste into the environment, it can be reused by partially replacing aggregates in concrete and mortar. Moreover, the effect of sand replacement by PET plastic waste was proven using different forms, and it was found that the shape and size can affect the concrete properties [18,19,20]. Saikia and de Brito [21] suggested that the replacement of sand with PET plastic waste resulted in a higher compressive strength than that of shredded PET plastic waste. However, the compressive strength was lower when the PET pellet form was used. Therefore, this research aimed to investigate the mechanical and physical properties of cement mortars using MIBA and PET bottle waste as a partial replacement for fine aggregate. The compressive strength, water absorption, and density were investigated. In addition, microstructures were examined using scanning electron microscopy. The results of this study can provide an alternative method for Si Chang Island to manage the incineration bottom ash and PET bottles as construction materials.

## 2. Materials and Methods

### 2.1. Raw Materials

The PET bottle waste used in this study was in the form of PET pellets made from recycled PET bottle waste. The PET pellets were obtained from Grand Siam Polymer Co., Ltd. The bottom ash used in this study was mixed ash consisting of fly ash and bottom ash and was obtained from an incinerator in the Si Chang municipality. Sand, MIBA, and PET pellets were sieved prior to use, and the particle size (less than 4.75 mm) was passed through a No. 4 sieve.

Sand, MIBA, and PET pellets were tested for aggregate properties, including particle size distribution, shape, surface texture, fineness modulus, water absorption, and specific gravity. Chemical composition was analyzed using an X-ray fluorescence spectrometer (Bruker model S8 Tiger). Mineral phases were identified using an X-ray diffraction instrument (Bruker AXS model S4 Pioneer, Karlsruhe, Germany). Microstructural characteristics were identified using a scanning electron microscope (Jeol JSM-6480LV). Only MIBA was analyzed for heavy metal leaching using the Toxicity Characteristic Leaching Procedure (TCLP) (USEPA, Washington, DC, USA, 1992).

### 2.2. Production of Mortar Specimens

The mix proportion was designed according to ASTM C109 and modified to achieve the target compressive strength at 40 MPa after 56 days of curing by immersion in water. As shown in Table 1, the binder/fine aggregate ratio was 1:3, and the water/cement ratio was 0.5 for all mixes. MIBA was used as a replacement for fine aggregate at 0%, 10%, 20%, 30%, and 40% *w*/*v*, and PET pellets were used as a replacement for fine aggregate at a replacement level of 0%, 10%, and 20% *w*/*v*. Twelve mortar specimens were prepared for each mix. Six specimens were used for compressive strength tests and another six for water absorption tests. The specimens from all mixes were tested for compressive strength and water absorption according to ASTM C39 and ASTM C64, respectively.

## 3. Results and Discussion

### 3.1. Raw Materials

#### 3.1.1. Fine Aggregate Properties

Sand, MIBA, and PET pellets (PET) were analyzed to investigate the particle size distribution, shape, surface texture, fineness modulus, water absorption, and specific gravity.

1. Particle Size Distribution

**Figure 1:**
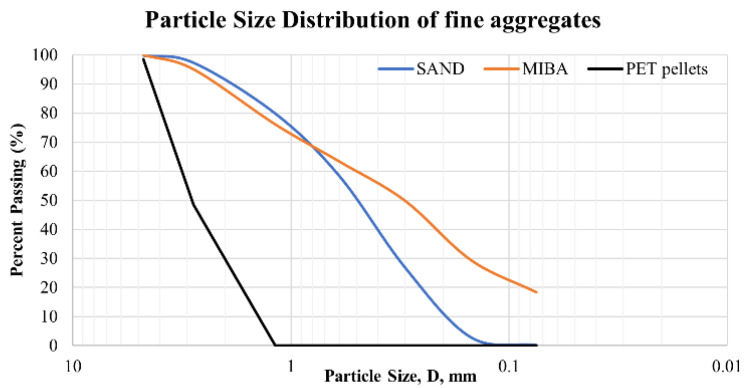
Particle size distribution of fine aggregates.

The particle size distribution was analyzed using the sieving method to determine the average particle size of raw materials (Figure 1). The particle size distribution results indicated that the PET pellets were larger than sand and MIBA. This is due to the fixation and size consistency of PET pellets, which were retained only on sieves No. 7 (2.8 mm) and No. 16 (1.18 mm). As a result, the D_50_ values for MIBA, sand, and PET pellets were 0.3 mm, 0.5 mm, and 3 mm, respectively.

2. Shape and Surface Texture

**Figure 2:**
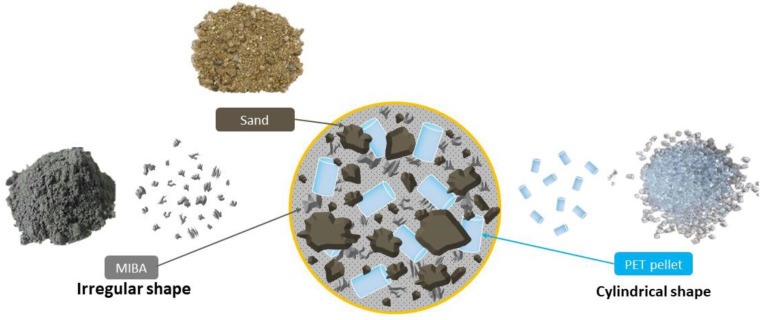
General appearances of sand, MIBA, and PET pellets.

Sand has different grain sizes and shapes and is rough and angular. MIBA is fine-grained, dry, and dark gray in color. In comparison, PET pellets are cylindrical with smooth surfaces and bright colors. The appearance of raw materials is shown in Figure 2.

3. Fineness Modulus

Sand, MIBA, and PET pellets have a fineness modulus (F.M.) of 2.35, 1.86, and 4.53, respectively. Increasing the F.M. can affect the compressive strength of the samples and improve it [21]. The water absorption of fine aggregates was analyzed according to ASTM C128. The water absorption of sand, MIBA, and PET pellets was 1.1%, 18.5%, and 0%, respectively. The PET pellet is a nonabsorbent material with a smooth, nonporous surface [22]. Meanwhile, the high-water absorption of MIBA requires more water, reducing the actual water–cement ratio [21].

The specific gravity of fine aggregates was analyzed according to ASTM C128. The results showed that the specific gravity of sand, MIBA, and PET pellets is 2.48, 1.27, and 1.28, respectively.

4. Characteristics of Fine Aggregates

#### 3.1.2. Chemical Composition

The chemical compositions of raw materials were analyzed using an X-ray fluorescence spectrometer (Bruker model S8 Tiger). As shown in Table 2, SiO_2_ (73.6%) and CaO (31.3%) are the major components of sand and MIBA, respectively. Chlorine (Cl) is also high in MIBA (5.22%). Due to improper waste incineration without efficient waste separation, the CI content can be inherited from PVC, chloride-contained plastics, and chloride salts in kitchen waste [23]. The chloride content in MIBA can corrode the reinforcing steel in concrete, causing the structure to collapse [24].

5. Mineral Phases

**Figure 3:**
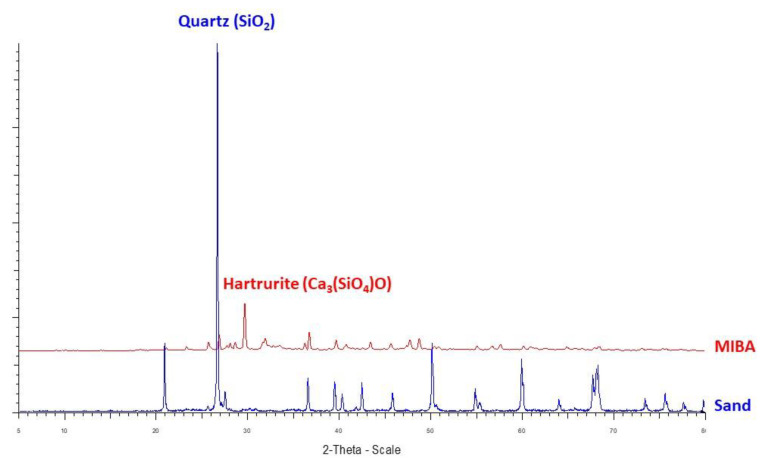
X-ray diffraction patterns of sand and MIBA.

The X-ray diffraction pattern of raw materials is shown in Figure 3. The major crystalline phases of sand are quartz (SiO_2_) and orthoclase (KSi_3_AlO_8_). The major crystalline phases of MIBA are synthetic hartrurite (Ca_3_SiO_5_) and wollastonite (CaSiO_3_), whereas the minor mineral phases are marcasite (FeS_2_) and pyrrhotite (FeS).

6. Microstructure and Elemental Composition of Raw Materials

**Figure 4:**
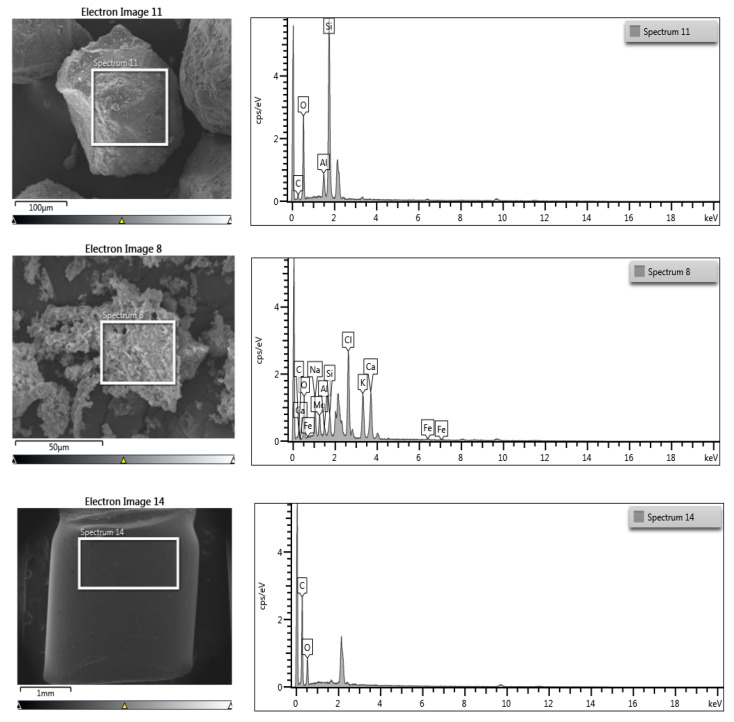
Microstructural characterization and chemical composition of raw materials.

The microstructure and elemental composition of raw materials are shown in Figure 4. The particle morphologies were observed at 50× resolution for sand and MIBA and 30× resolution for PET pellets. The results showed that sand had a rough and angular surface, while MIBA was noticeably smaller and more porous than sand, which was confirmed at the same magnification. However, the PET pellets were much larger than sand and MIBA and had a smooth non-angular surface, and were nonporous. In addition, these products have different elemental components. For example, Si and C are the major elements of PET and sand, respectively. On the other hand, MIBA has a relatively diverse composition consisting of several elements, with CaO being the most prominent.

7. Leaching of Heavy Metals from MIBA

Only MIBA was examined for the leaching of heavy metals, including Ba, As, Co, Cd, Fe, Cr, Mn, Cu, Se, Zn, Ni, and Pb, using the TCLP method. As shown in Table 3, the results were compared to the regulatory levels of the U.S. Code of Federal Regulations [25] and the soil quality standards of the Thai Pollution Control Department [26]. The heavy metal concentration of MIBA was within the maximum contaminant concentration for toxicity characteristics. As a result, MIBA can be classified as nonhazardous waste and used as a raw material in this study. Thus, the cement mortars made from MIBA did not require heavy metal leaching testing.

### 3.2. Mortar Specimens

#### Physical Properties of Mortar Specimens

1. General Appearance

**Figure 5:**
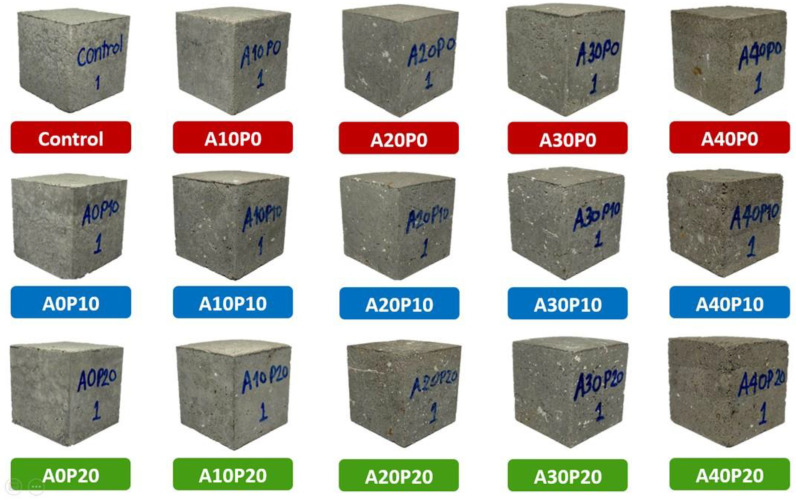
General appearance of mortar specimens.

As shown in Figure 5, the general appearance of mortar specimens differs slightly in those with high levels of MIBA. In addition, the mixture layers, surface roughness, and dryness can be observed compared to the control.

2. Compressive Strength

**Figure 6:**
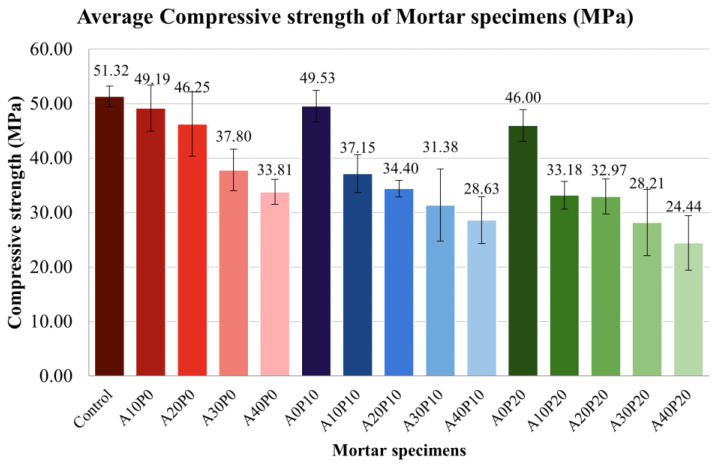
Average compressive strength of mortar specimens.

Figure 6 shows the compressive strengths of mortar specimens after 56 days. The results show that the compressive strength of each group decreased as the amount of MIBA used to replace sand increased. Four mortar specimens have compressive strengths greater than 40 MPa, but less than the control (51.32 MPa), as follows: A10P0 (49.19 MPa), A20P0 (46.25 MPa), A0P10 (49.53 MPa), and A0P20 (46 MPa).

**Figure 7:**
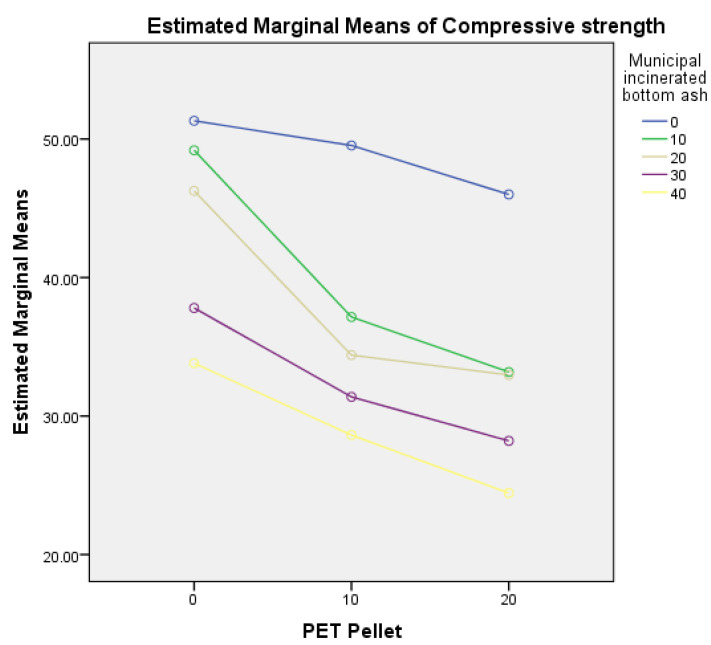
Estimated marginal means of compressive strength between PET pellets and MIBA.

As shown in Table 4, data analysis two-way ANOVA for compressive strength shows no interaction between PET pellets and MIBA, F (8, 60) = 1.897, *p* = 0.08. This demonstrates that the combination of PET pellets and MIBA has no significant effect on specimen compressive strengths. At the same time, the effects of PET pellets and MIBA have a p-value = 0.000, indicating that the amount of PET pellets and MIBA affects the compressive strength of the specimen at a significant level of 0.01. This can describe how different replacement levels of both materials affect the compressive strength. However, as shown in Figure 7, the profile plot of estimated marginal means of compressive strength between PET pellets and MIBA shows trends in the same direction.

3. Water Absorption

**Figure 8:**
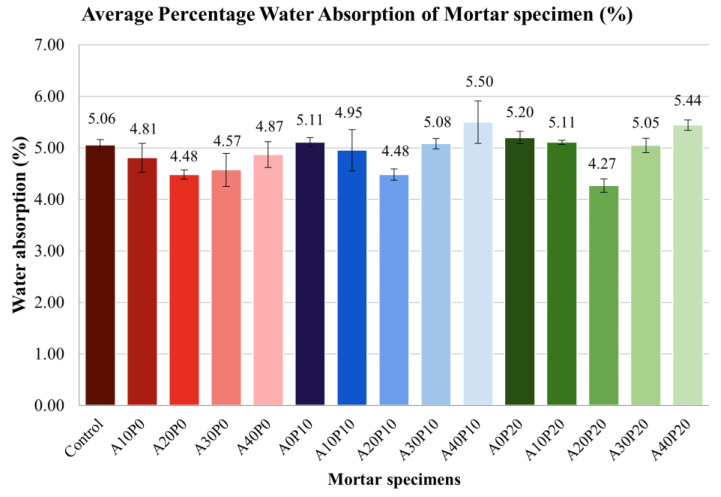
Average water absorption of mortar specimens.

Figure 8 shows the water absorption of mortar specimens. The results indicate that the highest water absorption rate was A40P10 (5.5%), followed by A40P20 (5.44%), whereas the lowest was A20P20 (4.27%). This experiment shows that using 20% MIBA caused the lowest water absorption for each group. In addition, among all ratios, 20% MIBA and 20% PET pellets have the lowest water absorption. It can be concluded that replacing sand with 20% PET pellets and 20% MIBA resulted in the best aggregate arrangement with the least porous matrix and less water absorption.

**Figure 9:**
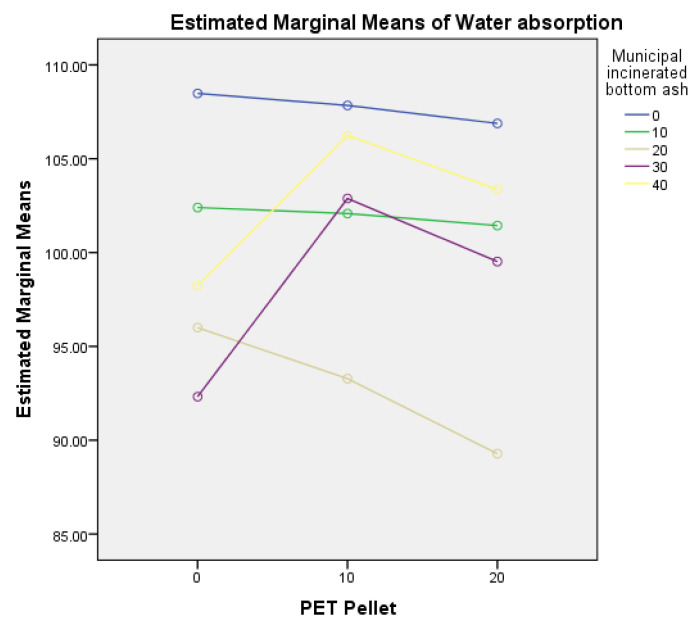
Estimated marginal means of water absorption between PET pellets and MIBA.

The data analysis of water absorption (Table 5) revealed an interaction effect between PET pellets and MIBA, F (8, 60) = 2.22, *p* = 0.004. This demonstrates that combining different levels of PET pellets and MIBA significantly impacts the specimen’s water absorption. Furthermore, the plot of estimated marginal means for different replacement levels of PET pellets and MIBA (Figure 9) shows that the use of 30% and 40% MIBA tends to differ from other scenarios.

4. Density

**Figure 10:**
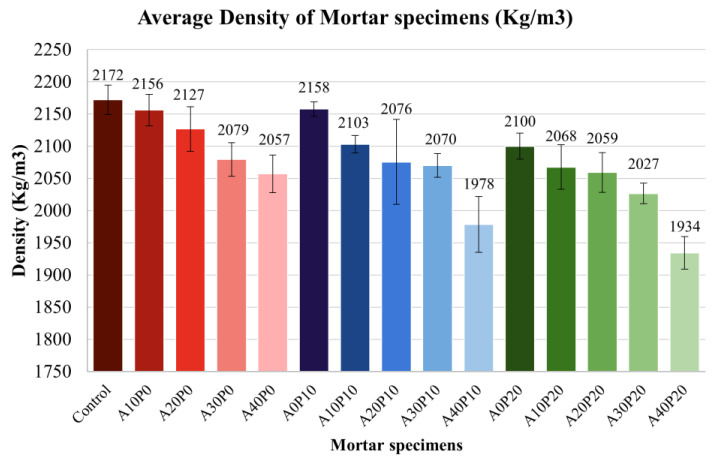
The average density of mortar specimens.

Figure 10 shows the density of mortar specimens. The tendency of density is similar to that of compressive strength, indicating that these two parameters are correlated. The control has the highest density (2172 kg/m^3^), followed by A0P10 (2158 kg/m^3^) and A10P0 (2156 kg/m^3^). On the other hand, A40P20 presented the lowest density (1979 kg/m^3^).

**Figure 11:**
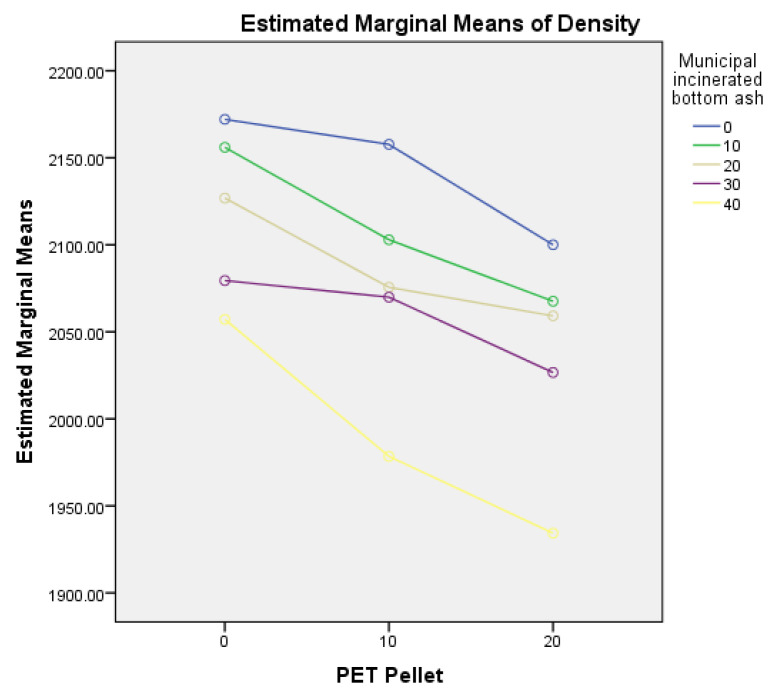
Estimated marginal means of density between PET pellets and MIBA.

The density analysis results (Table 6) revealed that the amount of PET pellets and MIBA significantly affected the specimen’s density. It can be observed that there is no interaction effect when using PET pellets with MIBA, F(8, 60) = 1.44, *p* = 0.2. The plots of the estimated marginal mean for different replacement levels of PET and MIBA (Figure 11) show a similar trend to the compressive strength plot.

5. Microstructure of Mortar Specimens

**Figure 12:**
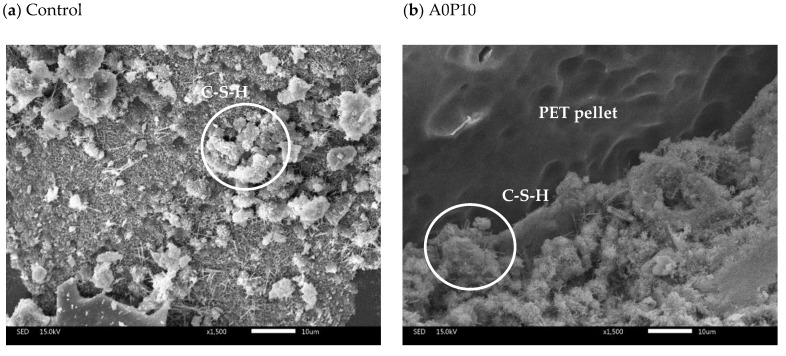

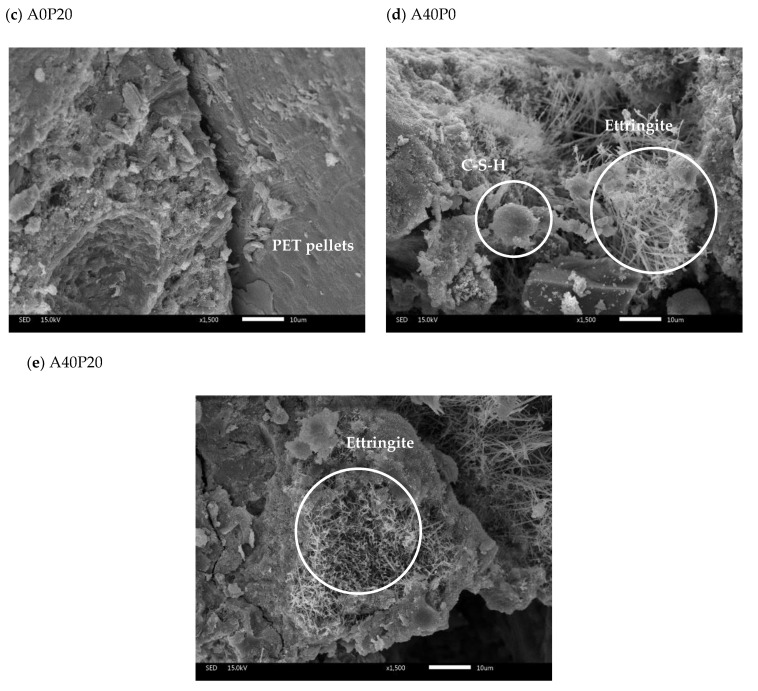
Microstructure of mortar specimens: control (**a**), A0P10 (**b**), A0P20 (**c**), A40P0 (**d**), and A40P20 (**e**).

The microstructure of mortar specimens was identified using a scanning electron microscope (IT300) at 1500× magnification. Only mortar specimens selected from the control (no waste), A0P10 (best performance), A0P20 (highest PET pellet replacement), A40P0 (highest MIBA replacement), and A40P20 (worst performance, most uses of both wastes) were analyzed. The results revealed calcium silicate hydrate (CSH) and ettringite on the surface of mortar specimens (Figure 12). Ettringite shows needle-like crystals resulting from the hydration reaction of tricalcium aluminate (C_3_A) as sulfate ions of gypsum reacted with water. On the other hand, CSH is formed by the hydration reaction of calcium silicates (C_3_S and C_2_S) with water. Therefore, CSH is like a gel and serves as a binder that connects the aggregates and provides strength to the specimens [27].

**Figure 13:**
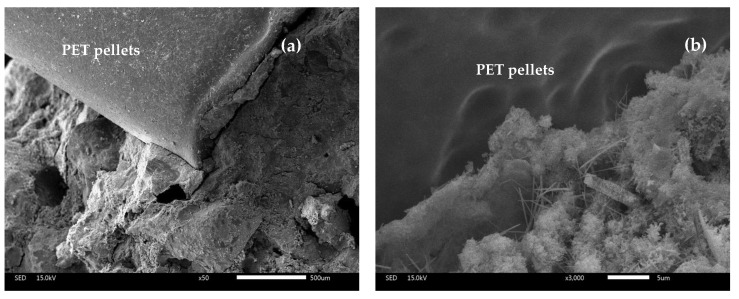
Microstructure of mortar specimens with PET pellets at 50× magnification (**a**) and 3000× magnification (**b**).

The microstructures of mortar specimens with PET pellets are shown in Figure 13a,b at 50× and 3000× magnifications, respectively. The smooth surface of PET pellets was found to be poorly connected with the mixture, resulting in more free space and voids.

The physical properties of mortar specimens showed a decline in compressive strength with the increased replacement of PET pellets and MIBA. da Silva and de Brito [22] studied the replacement of sand with two types of plastic aggregates: PET pellets and PET flakes. The results showed that PET pellets with a smooth surface, low specific surface area, and no water absorption decreased the W/C ratio and improved the workability of the mortar. However, a poorly connected matrix/aggregate results in high-porosity mortars. In addition, the mortar density is reduced linearly when using more plastic aggregate due to the lower density of the plastic aggregates than sand.

At the same time, Naran and Gonzalez [28] reported that single-plastic aggregates (PA) are hydrophobic, resulting in less water absorption. The excess water was used to coat and decrease the friction between particles. Moreover, the bonding between cement and PA is not strong and causes voids, resulting in a lower compressive strength and high-water absorption.

According to the morphology of MIBA, which is irregular in shape, rough surfaces and a high porosity cause a high water absorption. In addition, MIBA has a lower density than natural aggregates, causing a lower density of the specimens when increasing the replacement of sand [29].

Kunther and Ferreiro [30] studied the influence of the Ca/Si ratio on the compressive strength of the sample and found that the high Ca/Si ratio decreased the quantity of calcium silicate hydrates. In addition, the raw material analysis showed that MIBA had higher CaO than SiO_2_. Therefore, the Ca/Si ratio of mortar specimens increased when increasing the replacement levels of MIBA, decreasing the compressive strength. The Ca/Si ratio also affects the microstructure of the samples, increasing the compressive strength, reducing water absorption, and reducing microcracks [31].

A previous study of using reactive aggregate to produce concrete samples shows that the effects of the alkali–silica reaction on the compressive strength and elastic modulus of the samples can be observed after curing by immersion for 28 days, lowering the compressive strength and elastic modulus of concrete samples [32].

The relationship between density and compressive strength shows that mortar specimens with a high density had a high compressive strength. Increasing the sand replacement rate by lightweight waste results in a decrease in density. MIBA and PET pellets have a lower specific gravity than sand, with poor bonding between aggregates. Moreover, MIBA has a high CaO content. Hence, using more MIBA may increase the Ca/Si ratio. The results showed that the compressive strength decreased as the amount of CSH was reduced. On the other hand, the water absorption of mortar specimens is related to the porosity and the connection between aggregates. The results showed that using 20% MIBA with different levels of PET pellets provided the lowest water absorption for each group, and the trend was similar for each group. Therefore, it can be concluded that a 20% sand replacement by MIBA is a suitable ratio that leads to the lowest water absorption. The effect of water absorption is not directly related to compressive strength. However, as MIBA increased, the CSH formation decreased, resulting in poor aggregate connectivity, pore generation in the microstructure, and a decrease in compressive strength. However, the 10% and 20% replacement of MIBA produced different results. As a result of the diversity of MIBA sizes, substitution at 10% could result in poor alignment and the development of pores. However, at a 20% substitution, better results were obtained because the CaO content in MIBA was optimal.

## 4. Conclusions

This study aimed to investigate the feasibility of utilizing MIBA and PET plastic waste from Si Chang Island. Both wastes were used as partial substitutes for the fine aggregate in cement mortar production. The optimal ratio of cement mortar production was determined, and the properties of the cement mortars were examined. The following conclusions can be drawn from the results of this study:

1. The compressive strengths of mortar specimens cured for 56 days was greater than 40 MPa, and are as follows:
  - A0P10 with 10% sand replaced by PET pellets obtained a compressive strength of 49.53 MPa.
  - A10P0 with 10% sand replaced by MIBA obtained a compressive strength of 49.19 MPa.
  - A20P0 with 20% sand replaced by MIBA obtained a compressive strength of 46.25 MPa.
  - A0P20 with 20% sand replaced by PET pellets obtained a compressive strength of 46.00 MPa.
2. The properties of the mortar specimens showed that the amount of waste replaced by fine aggregate in the manufacture of cement mortar affected the reduced compressive strength and density of mortar specimens due to the poor bonding of aggregates in the mortar specimen matrix and low-density properties of the waste. However, mortar specimens with 20% sand replaced by PET pellets obtained the lowest water absorption.

The MIBA used in this study was classified as nonhazardous waste due to the amount of leaching heavy metals examined by the TCLP method not exceeding the regulatory levels of the U.S. Code of Federal Regulations and the soil quality standards of the Thai Pollution Control Department. In addition, this study showed that replacing 10% and 20% sand in cement mortar with only MIBA or only PET pellets could result in compressive strengths ranging from 46.00 MPa to 49.53 MPa. Based on these findings, the following future research topics were proposed for the alternative use of MIBA and PET bottles in building and construction work in Si Chang Island: an investigation of mechanical properties, such as tensile strength, elastic modulus, and stress–strain curves, as well as a life cycle assessment and economic feasibility.

## Figures and Tables

**Table 1:** Mortar formulations.

Name	Binder		Fine Aggregate		Water/Cement
	Cement	Sand (%)	MIBA (%)	PET Pellets (%)	
Control	100	100	0	0	0.5
A10P0	100	90	10	0	
A20P0	100	80	20	0	
A30P0	100	70	30	0	
A40P0	100	60	40	0	
A0P10	100	90	0	10	
A10P10	100	80	10	10	
A20P10	100	70	20	10	
A30P10	100	60	30	10	
A40P10	100	50	40	10	
A0P20	100	80	0	20	
A10P20	100	70	10	20	
A20P20	100	60	20	20	
A30P20	100	50	30	20	
A40P20	100	40	40	20	

Note: A refers to MIBA, and P is defined as PET pellets.

**Table 2:** Chemical composition of sand and MIBA.

Oxides	Content (wt.%)	
	Sand	MIBA
SiO_2_	73.6	17.0
Al_2_O_3_	4.72	4.22
K_2_O	2.96	3.03
Fe_2_O_3_	0.765	2.07
Na_2_O	0.266	3.57
TiO_2_	0.125	1.19
MgO	868 PPM	2.31
CaO	868 PPM	31.3
BaO	359 PPM	729 PPM
P_2_O_5_	345 PPM	1.96
Rb_2_O	184 PPM	60 PPM
ZrO_2_	141 PPM	156 PPM
SO_3_	105 PPM	3.04
MnO	87.4 PPM	875 PPM
SrO	34.7 PPM	402 PPM
PbO	27.4 PPM	300 PPM
Cl	Not detected	5.22
CoO	Not detected	Not detected
NiO	Not detected	81.6 PPM
CuO	Not detected	0.118
ZnO	Not detected	0.22
As_2_O_3_	Not detected	Not detected

**Table 3:** Leaching of heavy metals from MIBA.

Heavy Metal	MIBA (mg/L)	Regulatory Level (mg/L)	Soil Quality Standards (mg/kg)
Ba	0.638 ± 0.169	100.0	-
As	Not detected	5.0	3.9
Co	Not detected	-	-
Cd	0.003 ± 0.000	1.0	37
Fe	Not detected	-	-
Cr	0.228 ± 0.028	5.0	300
Mn	Not detected	-	1800
Cu	0.188 ± 0.058	-	-
Se	0.010 ± 0.000	1.0	390
Zn	0.013 ± 0.008	-	-
Ni	Not detected	-	1600
Pb	0.011 ± 0.003	5.0	400

**Table 4:** Compressive strength tests between PET pellets and MIBA effects. Test of Between-Subjects Effects. Dependent Variable: Compressive strength.

Source	Type III Sum of Squares	df	Mean Square	F	Sig.	Partial Eta Squared
Corrected Model	5282.550 ^a^	14	377.325	22.857	0.000	0.842
Intercept	106,129.133	1	106,129.133	6428.935	0.000	0.991
PET	1508.426	2	754.213	45.688	0.000	0.604
MIBA	3523.559	4	880.890	53.361	0.000	0.781
PET*MIBA	250.564	8	31.321	1.897	0.077	0.202
Error	990.483	60	16.508			
Total	112,402.165	75				
Corrected Total	6273.033	74				

^a^ R Squared = 0.842 (Adjusted R Squared = 0.805).

**Table 5:** Water absorption tests between PET pellets and MIBA effects. Test of Between-Subjects Effects. Dependent Variable: Water absorption.

Source	Type III Sum of Squares	df	Mean Square	F	Sig.	Partial Eta Squared
Corrected Model	2413.943 ^a^	14	172.425	9.764	0.000	0.695
Intercept	760,274.953	1	760,274.953	43,051.327	0.000	0.999
PET	123.614	2	61.807	3.500	0.037	0.104
MIBA	1835.554	4	458.889	25.985	0.000	0.634
PET*MIBA	454.775	8	56.847	3.219	0.004	0.300
Error	1059.584	60	17.660			
Total	763,748.480	75				
Corrected Total	3473.527	74				

^a^ R Squared = 0.695 (Adjusted R Squared = 0.624).

**Table 6:** Density tests between PET pellets and MIBA effects. Test of Between-Subjects Effects. Dependent Variable: Density.

Source	Type III Sum of Squares	df	Mean Square	F	Sig.	Partial Eta Squared
Corrected Model	294,711.711 ^a^	14	21,050.837	20.365	0.000	0.826
Intercept	323,728,515.6	1	323,728,515.6	313,176.841	0.000	1.000
PET	81,567.215	2	40,783.608	39.454	0.000	0.568
MIBA	201,231.402	4	50,307.851	48.668	0.000	0.764
PET*MIBA	11,913.094	8	1489.137	1.441	0.199	0.161
Error	62,021.543	60	1033.692			
Total	324,085,248.9	75				
Corrected Total	356,733.254	74				

^a^ R Squared = 0.826 (Adjusted R Squared = 0.786).

## Data Availability

The data presented in this study are available on request from the corresponding author.
